# Coccidioidomycosis in Europe: a systematic literature review of epidemiology, treatment and outcomes

**DOI:** 10.1093/jac/dkaf407

**Published:** 2025-11-06

**Authors:** Priya Shastri, Gurjinder Bains, Anne Santerre Henriksen, Andreas Karas

**Affiliations:** Medical Affairs, Shionogi B.V., 50 Eastbourne Terrace, London, W2 6LG, UK; Medical Affairs, Shionogi B.V., 50 Eastbourne Terrace, London, W2 6LG, UK; Medical Affairs, Shionogi B.V., 50 Eastbourne Terrace, London, W2 6LG, UK; Medical Affairs, Shionogi B.V., 50 Eastbourne Terrace, London, W2 6LG, UK

## Abstract

**Objectives:**

Coccidioidomycosis (cocci) is a rare fungal disease with clinical manifestations ranging from asymptomatic to life-threatening severe pulmonary or disseminated infection. It is endemic to parts of the southwestern USA, Mexico and South America. Tourism and migration have led to cocci occurrences across Europe among those arriving from endemic areas. The pan-European epidemiology of cocci has not previously been systematically reviewed.

**Methods:**

A systematic literature review was conducted with no limits for publication year, article type and language. We initially identified 797 records, and 61 publications were retrieved in accordance with the inclusion criteria. We identified 55 case reports and six case series, which spanned 20 countries.

**Results:**

Overall, 197 cocci cases were reported in the literature between 1948 and 2022, with the highest number being from Spain (96 cases), Russia/USSR (35) and France (20). Ninety-eight cases were due to migration, 39 infections were travel-related, and 5 were donor derived. Infection sites included pulmonary (39%), CNS (10%) and bone/joint (1%). Infection site was not reported for 36% of the cases. Most patients had a differential diagnosis, resulting in delayed treatment. Fluconazole (27%) or amphotericin B (19%) were most frequently prescribed as first-line antifungal therapy. Mortality and stable disease rates were 7% and 6%, respectively, with higher rates in immunocompromised patients (24% and 14%).

**Conclusions:**

Clinicians should consider cocci in the differential diagnosis of patients with pneumonia and/or meningitis symptoms who have travelled from endemic regions.

## Introduction

Coccidioidomycosis (or ‘cocci’, also colloquially referred to as Valley fever) is an airborne infectious disease caused by the dimorphic fungi *Coccidioides immitis* and *Coccidioides posadasii*. Previously cocci has been considered as endemic to southwestern USA (California and Arizona) and Mexico as well as areas in South America.^1,2^ However, climate change and population growth have led to a broadening in the endemic range of the fungus leading to increases in prevalence. The climate-driven expansion of endemic regions is expected to continue thereby contributing to further increasing prevalence of the disease.^3^ Increasing intercontinental travel has coincided with reported cases in Europe among returning tourists or those migrating from endemic regions, highlighting the need for vigilance beyond endemic areas.

Cocci is not generally contagious via human-to-human airborne transmission; however, human–human transmission in solid organ transplant via undetected infected donor graft is occasionally reported.^4–7^ The disease is primarily contracted through the inhalation of spores present in the soils of endemic regions and can affect both immunocompetent and immunocompromised individuals. The clinical manifestations in humans are diverse. Approximately 60% of cases are asymptomatic. Of the remaining ∼40% that present with symptoms, most have pulmonary disease ranging from self-limiting influenza-like illness to pneumonia. About 10% of these symptomatic patients develop severe complications, and roughly 1% of all cases progress to extrapulmonary dissemination^8–12^ Sites of dissemination vary and include skin (common), lymph nodes, bone and the CNS but can affect virtually any organ system.^13^ Treatment choice is dependent on the severity of disease and immune status. In patients without overt immunosuppressing conditions and an uncomplicated coccidioidal pneumonia or those who have mild non-debilitating symptoms, or who have improved or resolved clinical illness by the time of diagnosis, education, close observation and supportive measures are recommended. For patients with extensive pulmonary involvement with concurrent diabetes mellitus, frailty due to age or comorbidities, or certain ethnic backgrounds deemed to be at greater risk of complications, oral therapy with the triazole antifungal agent fluconazole is the preferred treatment for non-pregnant adults, with itraconazole the second-line option. For other complications, including cavitary disease, osteomyelitis, or those with CNS disease, treatment recommendations including when to use amphotericin B and/or surgical intervention are described by the IDSA.^10^ Of note, although oral fluconazole is the standard of care for CNS infections currently, all triazole therapy must be continued lifelong due to the inability of current treatment options to eradicate disease at this site. Patient inability to tolerate triazoles for extended periods and ongoing neurological symptoms from disease are an unresolved concern.^1–3^

In 2022, the WHO developed the first fungal priority pathogen list. Fungal pathogens were prioritized according to a set of criteria based on public health importance, incidence of antifungal resistance, knowledge gaps on disease burden, surveillance and unmet R&D needs. Amongst the selected pathogens, *Coccidioides* spp. were classified as medium priority, emphasizing the need for treatment R&D and innovation.^14^

The diagnosis of cocci is challenging and relies upon initial suspicion based on clinical and epidemiological characteristics. Clinical characteristics in addition to basic infection and inflammatory markers are non-specific and include: influenza-like signs and symptoms; abnormal lung findings on chest imaging; involvement of bones, joints or skin by dissemination; skin symptoms such as erythema nodosum or erythema multiforme rash or single or multiple lesions; meningitis or involvement of viscera or lymph nodes; or, finally, abscess, granuloma or lesion in other body systems.^15^ For definitive diagnosis, the core approach is detection of antibody via enzyme immunoassays (EIAs), supplemented by direct microscopy or histological visualization of spherules and fungal culture. Culture yield is low, especially from CSF, but if positive requires immediate transfer to a biosafety level 3 (BSL3) laboratory before any manipulation.^16^

Currently, epidemiology, treatment practice and outcomes of coccidioidomycosis across Europe are unknown. Therefore, a systematic review of the literature was conducted.

## Methods

A systematic literature review was conducted using PubMed, EMBASE and Google Scholar pertaining to the research question: What is the epidemiology, treatment practice and outcomes of coccidioidomycosis in Europe?

**Figure 1.**
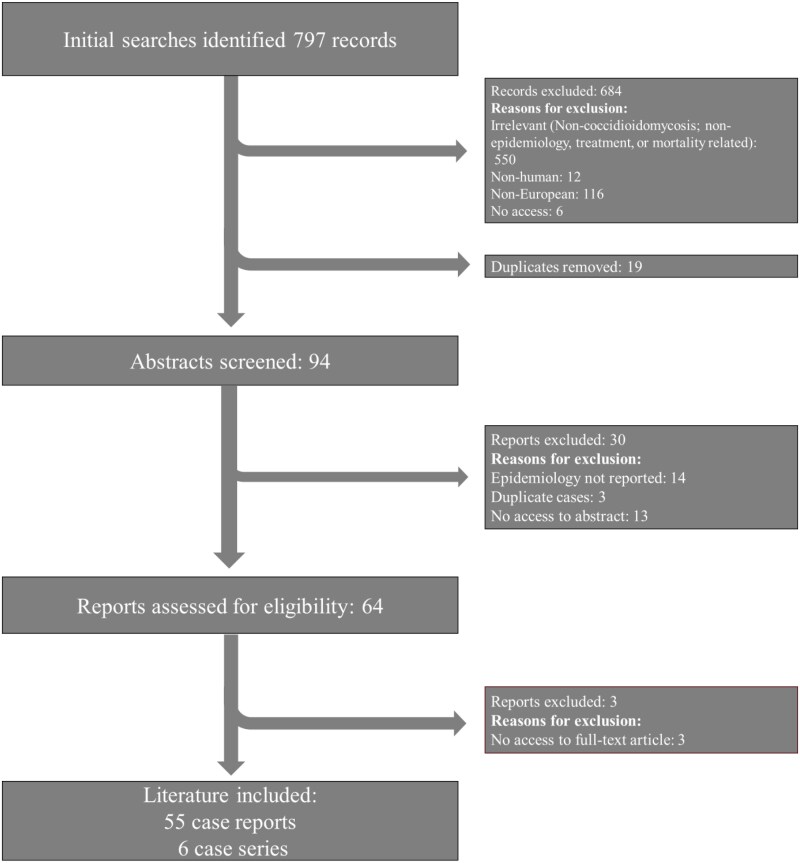
PRISMA flow chart of article selection.

The search string was formed using the following terms ‘coccidioidomycosis’ OR ‘acute pulmonary coccidioidomycosis’ OR ‘chronic pulmonary coccidioidomycosis’ OR ‘disseminated coccidioidomycosis’ OR ‘progressive coccidioidomycosis’ OR ‘acute primary coccidioidomycosis’ OR ‘*Coccidioides immitis*’ OR ‘*Coccidioides posadasii*’ AND ‘Europe’. Due to the rarity of the condition in Europe, no limits were set for publication year, article type and language. Additionally, relevant citations within each publication were included. Afterwards, publications were screened accordingly via inclusion and exclusion criteria (Figure 1).

The type of articles included consisted of case reports and epidemiology review papers. All cases must have been diagnosed and/or have had a positive culture and/or serology and/or histology for cocci, and included those testing positive during autopsy. Epidemiology reports were included with or without the description of treatments given or outcomes to provide a comprehensive overview on the number of cases within Europe. Such methods followed a protocol in accordance with the Preferred Reporting Items for Systematic Reviews and Meta-Analysis Protocols (PRISMA-P).

The literature search was conducted on 11 June 2024, identifying a total of 797 records, from which 94 abstracts were screened and 61 publications were retrieved (Figure 1), dating from 1948 to 2022. Of the 61 publications, 55 were case reports and six were case series.^4–7,17–73^

## Results

**Figure 2.**
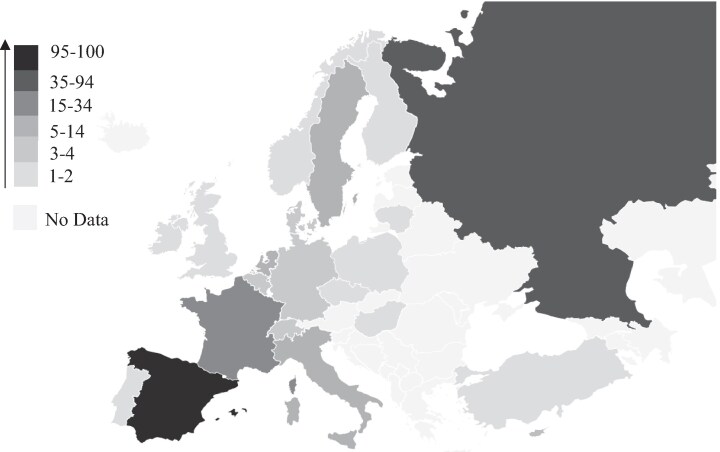
Heat map illustrating the reported incidence of coccidioidomycosis across Europe.

Sixty-one publications with 197 cases were included in this review (Table S1; available as Supplementary data at *JAC* Online). A total of 20 countries in Europe reported at least one case of cocci, as shown in Figure 2. Spain reported the highest number of cases (96), followed by Russia/USSR (35) and France (20) (Table 1 and Figure 2). France had the highest number of individual publications (Table 1).

**Table 1.** Country breakdown by number of publications and cases

Country	Number of publications	Number of cases
Belgium	2	2
Czech Republic	1	1
Denmark	2	2
Finland	1	2
France	12	20
Germany	4	4
Hungary	2	2
Italy	7	7
Lithuania	1	1
Netherlands	6	6
Norway	1	1
Poland	2	2
Portugal	1	1
Republic of Ireland	2	2
Russia/USSR	1	35
Spain	3	96
Sweden	5	5
Switzerland	4	4
Turkey	2	2
United Kingdom	2	2
*Total*	*61*	*197*

The analysis of patients’ characteristics revealed a higher percentage of male patients [94 (48%)] compared with female patients [52 (26%)]. The observed age range was 6–77 years, with a median of 46 (Table 2). The most common site of infection was pulmonary [77 (39%)], the primary described pulmonary presentation being nodular [14 (18%)] followed by CNS [19 (10%)] (Table 2). A large proportion of infection sites were not described [71 (36%)].

**Table 2.** Patient characteristics

Characteristic	Patients included in review, *n* (%)
Female	52 (26.4)
Male	94 (47.7)
Unknown gender	51 (25.9)
Age range (median), y	6–79 (46)
*Site(s) of infection*	
Pulmonary infection	77 (39.1)
Presentation unknown	49 (63.6)
Pulmonary nodule	14 (18.2)
Mixed presentation^a^	6 (7.8)
Pulmonary infiltrate	5 (6.5)
Pulmonary cavity	2 (2.6)
Pulmonary abscesses	1 (1.3)
Unknown	71 (36.0)
CNS infection	19 (9.6)
Non-CNS disseminated infections	14 (7.1)
Skin infections	9 (4.6)
Bone/joint infection	2 (1.0)
Lymphatic	2 (1.0)
Kidney infection	1 (0.5)
Liver infection	1 (0.5)
Pelvic	1 (0.5)
*Underlying risk factors/disease*	
Neoplasia	13 (6.6)
HIV/AIDS	7 (3.6)
Other immunosuppressive disorders	6 (3.0)
End-stage chronic kidney disease	1 (0.5)
Hepatic cirrhosis	2 (1.0)
Immunocompromised	2 (1.0)
Immunosuppressive therapy	1 (0.5)
Solid organ transplant	5 (2.5)
Haematological malignancy	3 (1.5)
Autoimmune disease	3 (1.5)
Bacterial infection	8 (4.1)
Other^b^	16 (8.1)
None	92 (46.7)
Undescribed	44 (22.3)

^a^Mixed presentation of various pulmonary characteristics listed.

^b^Other includes (*n*): diabetes (1), previous *in vitro* fertilization (IVF) patient (1), mitral valve prolapse and atrial fibrillation (1), tonsillitis and pregnant (1), coronary bypass and cigar smoker (1), spondyloarthritis with anterior uveitis, obesity and hypertension (1), background of benign prostatic hypertrophy only with mild impairment of liver function (1), adrenal insufficiency and knee replacement (1), Sweet syndrome (1), smoker (4), acute unilateral ophthalmoplegia (1), knee replacement (1), previous chronic lymphatic leukaemia and smoker (1).

Of the total 197 patients, 92 (47%) had no known underlying risk factors, 44 (22%) were undescribed, and 37 (19%) patients had an underlying disease associated with immunosuppression (Table 2). The most common underlying risk factors were neoplasia [13 (7%)], followed by HIV/AIDS [7 (4%)] (Table 2).

The acquisition of cocci was evaluated and it was found that a substantial proportion of patients had previously travelled or migrated from an endemic area. Ninety-eight infections followed migration from southwestern USA and parts of South America. Fifty-one cases did not describe how the infection was acquired. Forty infections were travel-related where patients had previously travelled to the USA, particularly Arizona [12 (31%)] as well as South America (Venezuela, Argentina and Colombia). Eight of the reported cases had never visited an endemic country. Of these, five were donor-derived cases of infection/graft-associated infection,^4–7^ two infections were occupationally acquired in a laboratory due to working in healthcare/research,^17,29^ and one patient had no reported travel history to a known endemic area.^37^

Pharmacological treatment practices and other interventions used were described in 47 (80%) publications accounting for 49 (25%) patients (Table 3). Thirty-seven (76%) patients were treated with effective monotherapy or surgery, of which 26 (70%) were treated with an antifungal, 9 (24%) had surgical intervention, and 2 (5.4%) were treated for symptoms only. Six (12%) patients had combination therapy with an antifungal, and four (8%) were able to spontaneously resolve their infection without intervention or surgery (Table 3). From those on monotherapy, a total of eight different first-line treatments were used to treat cocci. Fluconazole was the most commonly prescribed antifungal [10 (27%)] followed by amphotericin B [7 (19%)]. Half of the patients treated with fluconazole were unable to resolve their infection or required indefinite therapy, and five patients failed on amphotericin B (Table 3). After first-line therapy, eight patients went on to have sequential antifungal treatment, and two had surgical intervention; four patients needed three sequential therapies, and one patient needed up to five treatments (Table 3). For those on concomitant therapies, the antifungal combination of a triazole and amphotericin B was the most common (Table 3). Prior to antifungal or surgical intervention, 12 (24%) patients were treated with an antibacterial agent due to suspicion of TB or other bacterial infection;^7,20,24,27,31,45,51,53–55,62,67^ in addition, one patient was exclusively treated with an antibacterial.^25^ Another patient was treated with chemotherapy due to a history of Hodgkin’s lymphoma. The patient presented with flu-like symptoms after travelling to Arizona, hence a possible recurrence of Hodgkin’s disease was considered.^30^

**Table 3.** Known treatment practices from the included literature

Treatment type	*n* (%)
Unknown	148/197 (75.1)
Known^a^	49/197 (24.9)
Monotherapy^a^	39/49 (79.6)
Effective therapy^b^	37/39 (94.9)
Combination therapy	6/49 (12.2)
None	4/49 (8.2)
*First-line effective therapies (monotherapy)*	37/49 (75.5)
Fluconazole	10/37 (27.0)
Failed and switched treatment	3/10 (30.0)
Stable disease/life-long treatment	2/10 (20.0)
Surgical intervention	9/37 (24.3)
Amphotericin B	7/37 (18.9)
Failed and switched treatment	3/7 (42.9)
Stable disease/mortality	2/7 (28.6)
Itraconazole	6/37 (16.2)
Stable disease	1/6 (16.7)
Switched to amphotericin B	1/6 (16.7)
Symptomatic treatment	2/37 (5.41)
Stable disease	1/2 (50)
Posaconazole	1/37 (2.70)
Clinical resolution	1/1 (100)
Ketoconazole	1/37 (2.70)
Caspofungin	1/37 (2.70)
Switched to a triazole	1/1 (100)
*Second-line effective therapies (monotherapy)*	10/37 (27.0)
Surgical intervention	2/10 (20)
Itraconazole	2/10 (20)
Relapse	1/2 (50)
Fluconazole	1/10 (10)
Switched to amphotericin B treatment	1/1 (100)
Amphotericin B	3/10 (30)
Switched to triazole	1/3 (33.3)
Voriconazole	1/10 (10)
Failed leading to mortality	1/1 (100)
Ketoconazole	1/10 (10)
*Third-line effective therapies (monotherapy)*	4/37 (10.8)
Posaconazole	1/4 (25)
Itraconazole	1/4 (25)
Amphotericin B	2/4 (50)
Switched to a triazole	1/4 (50)
*Fourth-line effective therapies*	1/37 (2.7)
Voriconazole	1/1 (100)
Switched to amphotericin B treatment	1/1 (100)
*Fifth-line effective therapies*	1/37 (2.7)
Amphotericin B	1/1 (100)
Recommended life-long fluconazole therapy	1 (100)
*Combination therapies*	6/49 (12.2)
Triazole and amphotericin B	2/6 (33.3)
Antibiotics and antifungal	2/6 (33.3)
Subsequently on antifungal monotherapy	2 (100)
Adjunctive surgery and ketoconazole	1/6 (16.7)
Adjunctive surgery and amphotericin B	1/6 (16.7)

^a^Includes patients treated with antibiotics.

^b^Effective therapy as defined in the article reviews includes antifungal, surgical, and symptomatic treatment.

An overall mortality of 7% (14/197) was observed, and 6% (11/197) of patients reported stable disease. However, higher mortality was observed in immunosuppressed patients [9/37 (24%)] compared with those who were immunocompetent [4/116 (3%)] (Table 4). Similarly, morbidity was higher in immunosuppressed patients [5/37 (14%)] compared with immunocompetent patients [6/116 (5%)]. When broken down by infection site, immunosuppressed patients with a CNS infection had the highest mortality rate [3/3 (100%)] followed by pulmonary infections with 50% (3/6) mortality. Of the five patients with donor-derived cocci, three (60%) did not respond to antifungal therapy leading to mortality,^5–7^ one (20%) had an ongoing illness,^4^ and one (20%) patient had successful treatment.^6^

**Table 4.** Mortality and morbidity of patients from the literature according to immune status, not including patients that were lost to follow-up

All patients (*N* = 197)	Mortality, *n*/*N* (%)	Stable disease, *n*/*N* (%)
Overall	14/197 (7.1)	11/197 (5.6)
*Immunosuppressed patients, n*		
Overall (37/197) 18.8%	9/37 (24.3)	5/37 (13.5)
Pulmonary infection (6)	3/6 (50.0)	2/6 (33.3)
Donor-derived (3)	2/3 (66.7)	1/3 (33.3)
CNS infection (3)	3/3 (100)	N/A
Donor-derived (1)	1/1 (100)	N/A
Unknown location (25^a^)	3/25 (21.43)	N/A
*Immunocompetent patients, n*		
Overall (116/197) 58.9%	4/116 (3.4)	6/116 (5.2)
Pulmonary (33)	N/A	2/33 (6.1)
Unknown location (79^a^)	4/79 (5.1)	4/79 (5.1)
*Unknown underlying disease, n*		
Overall (44/197) 22.3%	1/44 (2.3)	N/A

^a^Includes data from the Spanish study by Molina-Morant *et al.*,^70^ which did not break down infection site according to immune status (71 immunocompetent and 23 immunocompromised patients).

## Discussion

This systematic review of coccidioidomycosis in Europe shows that incidence is rare, but the true incidence might be higher than described here given that only published cases were reviewed. Cocci should be considered when a relevant clinical picture and travel history are present. Here we discuss the unmet needs of cocci patients in Europe.

### Epidemiology

With 20 European countries having reported at least one case, an awareness of cocci is crucial, particularly for those treating immunocompromised patients. Spain reported the highest number of cocci infections. This is not surprising given the historic ties between the Iberian Peninsula and South America, which have fostered significant migration and travel between the regions. These strong connections likely contribute to the increased incidence of cocci observed in Spain.

In the future, global warming could potentially lead to the expansion of endemicity in the USA, particularly into northern states such as Washington.^16,74,75^ Patients reported in this review did not exclusively acquire cocci from known endemic regions, indicating that infections have also occurred in non-endemic areas. This may be related to factors such as rodent movement and/or climate change.^76^

### Patient characteristics

In Europe, the patient population was predominantly male, immunocompetent, and with a median age of 46. A large proportion of patients from the literature also presented with a pulmonary infection, which is the primary manifestation of cocci, and immunosuppressed patients were more likely to develop a complicated infection leading to mortality.^77,78^

Unlike in the USA, a median age of 46 was observed, which is younger than the typical patient population.^79^ Immunocompetent patients had a higher reported incidence in Europe, which is in line with the endemic population who contract cocci.^76^ This review suggests that cocci is likely to affect the younger and immunocompetent population in Europe, likely due to a higher frequency of younger people travelling to endemic areas and engaging in recreational activities such as hiking or for vocational purposes such as military training.^21,62^

### Cause and diagnosis

The majority of cases were acquired by migrants or returning travellers—the patient’s travel history played a crucial role during workup of diagnosis. In this review, 13 patients (27%) were initially treated with antibiotics, particularly those presenting with a community-acquired coccidioidal pneumonia, and were treated as TB or disease of bacterial aetiology.^7,20,24,25,27,31,45,51,53–55,62,67^ A study in southern California conducted in 2011 found that 70% of patients received antibiotics in the first 3 months prior to a positive cocci test.^80^ A clear unmet need in the diagnosis of cocci is apparent even in endemic regions. Therefore, awareness of cocci even in endemic regions requires improvement for patients to receive earlier appropriate therapy. A previous study from the USA analysed travel-related mycoses, such as histoplasmosis, paracoccidioidomycosis, coccidioidomycosis and blastomycosis, and stated that systemic endemic mycoses should be considered as potential travel-related infections in non-migrant international travellers.^81^

In certain instances, cocci was acquired without the patient having visited an endemic area. In some instances, this occurred in transplant patients where the donor had previously visited an endemic area resulting in the transmission of cocci to an immunocompromised host. A previous study analysed the occurrence of donor-derived fungal infections and infections post-transplantation across the USA over a 5 year period, and reported a total of nine cocci infections. The overall conclusion was that such findings are uncommon, but the duration of risk extends for years post-transplantation.^82^ In Europe such cases were also rare as only five were reported between 2009 and 2022. In a global evaluation of 34 cases of cocci post-transplant, two-thirds of patients were alive and healthy post follow-up.^83^ The outcomes from both papers describe a low mortality in this population; however, in our review only one patient was able to resolve their infection on antifungal therapy, which was recommended to continue indefinitely. Therefore, donor travel history that also considers endemic fungal pathogen exposure should be incorporated into routine donor-host risk assessment in Europe.^4,6^

In two instances our review reported cocci infections due to occupational exposure in the laboratory and hospital. A single inhaled spore is sufficient to cause infection from clinical specimens, and so in the USA the CDC have formulated prevention guidelines for research workers to handle samples in a BSL3 facility.^84^ In Europe there are currently no practical guidelines on handling non-endemic infections in this environment, despite *Coccidioides* spp. being classed as a group 3 hazard.^85–87^ Two studies reported safety measures taken post-exposure such as the shutting down of a laboratory for decontamination and the monitoring of researchers for cocci infection after handling samples.^7,62^ Hence, European guidelines for laboratory safety procedures should consider non-endemic infections to prevent future occurrences. Also, consideration should be given to make cocci a reportable illness in Europe due to the risk of lab infections and its potential as a bioterrorism agent.^88^

Test availability in Europe is limited due to the extreme rarity of cocci, which can lead to a delay in diagnosis and treatment. Antibody testing is the most important modality, but generally only available in reference labs in Europe, and gaining/maintaining accreditation for such tests is challenging when cases are so rare. Also, as a BSL3 laboratory is required, this limits the availability of testing across Europe. Such difficulties were described in two case reports where samples were sent internationally to identify *Coccidioides* spp. for diagnosis.^41,72^

### Treatment

The majority of treatment practices from the review were unknown due to aggregated epidemiological data which did not specify them. In the reported data, the majority of clinicians initiated fluconazole therapy in line with treatment guidelines for cocci.^10^ However, variable efficacy was observed as 40% (4/10) of patients failed treatment. For those treated with amphotericin B in this review, 71% of patients failed to clear the infection; in addition the formulations of amphotericin B were not specified in the majority of papers. This is likely due to the age of some papers and is a limitation of the current study. Amphotericin B is usually reserved for those who fail initial therapy after one or more high-dose oral azoles, or in special at-risk populations with very severe and/or rapidly progressing disease, and is given either IV or via the intrathecal route where there is CNS disease.^10^ An *in vivo* rabbit cocci meningitis model has suggested superior efficacy of IV liposomal amphotericin B compared with conventional IV amphotericin B or oral fluconazole.^89^

Treatment variability was observed suggesting uncertainty in treating cocci in Europe. Eight different interventions were initiated first-line of which only three coincided with the IDSA guidelines.^10^ Treatments such as amphotericin B in immunocompetent patients, ketoconazole—which is no longer a recommended therapy^76^—and caspofungin were prescribed as a first-line therapy. Antibiotic treatments were the second most initially prescribed therapy due to suspicions of TB or bacterial infection.

This was further emphasized by the number of sequential treatments: 27% (*n* = 10) of patients with a known treatment needed sequential therapy due to the inability to treat their infection. One case report in particular described a paediatric transplant patient requiring five sequential therapies due to the emergence of re-infection following an organ transplant.^6^ With current available treatments, at-risk populations are recommended to receive antifungal therapy for at least 6–12 months and in some cases indefinitely.^10^ As cocci is largely unknown in Europe there is a greater chance of inappropriate therapy; from these data, treatment practice for cocci in Europe is uncertain and displays variable efficacy.

### Outcomes

The majority of patients with cocci were successfully treated or had spontaneously resolved their infection. However, an increased mortality was observed in immunosuppressed patients, particularly those with CNS and pulmonary infection, which is the primary clinical manifestation. The immunocompetent group had no fatalities whereas the immunosuppressed had a mortality rate of 50% (3/6). Within the donor-derived subgroup, mortality was even higher [60% (3/5)]. With higher mortalities being reported in these groups, pre-transplant screening of organ donors is proposed, for a quick diagnosis and effective treatment in this setting.^90^

Immunosuppressed and immunocompetent groups reported similar numbers (5 [14%] and 6 [5%], respectively) of relapse. These patients include those with disseminated/chronic infections who remained on indefinite treatment despite clinical resolution due to high relapse rates seen in certain presentations of cocci.^91^ Care must be taken with long-term use of azoles due to various important adverse events, which may eventually lead to the discontinuation of treatment.^92^

### Limitations

This is a review of the current literature and as such is subject to the inherent biases of such work, including reporting bias and those inherent in the original work. We limited this through a systematic methodology that applied inclusion/exclusion criteria prior to conducting the literature search (Figure 1). Another limitation to this study was the proportion of reports that did not disclose infection site or treatment provided, limiting the data extracted. This was mitigated by including all patients where appropriate in the analysis and including unknown data rates such as infection sites in the mortality assessment. Although there are no nationwide databases in Europe that collect reports of cocci (as it is not a notifiable disease) future research could use mycology reference laboratory data to gain a clearer understanding of incidence within countries. This would address some of the limitations of using solely the literature to describe epidemiology.

### Conclusion

Coccidioidomycosis is not endemic to regions outside of the Americas, thus reported cases are rare, limiting the strength of any conclusions from this European-focused study. However, cases where they do occur can be life threatening, particularly in immunocompromised patients, specifically those with CNS involvement, mirroring the situation in endemic regions. Within Europe coccidioidomycosis appears to be difficult to diagnose due to limited knowledge of the disease and delays in obtaining a coccidioidal test, such as an EIA, particularly in donor-derived cases, leading to delayed treatment. Therefore, clinicians should consider including cocci as part of a differential diagnosis when there is a relevant travel history and also include this in pre-transplant workup for donors. Overall, increased accessibility of intercontinental travel as well as a possible change in epidemiology has made endemic infections such as cocci more important, and the understanding of diagnosis and treatment pathways is crucial to potentially reduce morbidity and mortality of high-risk patients in non-endemic areas.

## Supplementary Material

dkaf407_Supplementary_Data

## Supplementary data

Table S1 is available as Supplementary data at *JAC* Online.
