# Supplementary material for: Coccidioidomycosis in Europe: a systematic literature review of epidemiology, treatment and outcomes
Source: J Antimicrob Chemother. 2025 Nov 6;81(1):dkaf407. doi: 10.1093/jac/dkaf407 (PMC12802897; doi:10.1093/jac/dkaf407)
Supplement: dkaf407_Supplementary_Data [file dkaf407_supplementary_data.docx]

Supplementary Table 1: Summary of publications that were included in the review

| Country | Publication type | Summary | Reference |
| --- | --- | --- | --- |
| France | Case report | 58-year-old male transplant recipient contracted CM from the donor who previously travelled to Arizona. The recipient developed pulmonary CM and was treated with itraconazole and surgical repair of gastroesophageal reflux. From the report the patient has stable disease with antibody testing for *C. immitis* every 6 months with positive serology. | **4** |
| Portugal | Case report | 62-year-old male renal transplant patient with cerebral involvement. Treated with AmB lipid complex (4.5 mg/kg/day; 300 mg/ day) prior to culturing of the strain, then IV voriconazole was initiated (360 mg twice a day in the first 24 h and later 240 mg twice a day) was added to the antifungal regimen - was on therapy for around 2 months. The patient was unable to resolve his infection. | **5** |
| France | Case report | Donor-derived infection. Donor had a 12-mm calcified pulmonary nodule with subsequent infection in two transplant recipients.  Case 1: 6-year-old female in France receiving liver transplant. The girl was treated with caspofungin 2 days after transplant, afterwards IV fluconazole for 3 weeks, and then AmB, for 3 months, she developed a fever and was on voriconazole for 72 hours and then back on AmB for 55 days. She was recommended life-long fluconazole therapy.  Case 2: adult male lung transplant recipient was treated with fluconazole for 8 days and then switched to AmB. He was unable to resolve his infection. (Age not described) | **6** |
| France | Case report | 49-year-old male lung transplant recipient acquired CM through donor who had previously worked in Peru for 3 months. On return to France the donor had symptoms of fatigue, diarrhoea, nausea, weight loss, but CM was not diagnosed and donor passed away. Post lung transplant the recipient presented with a left lobar pneumonitis and a month after transplant necrotic sutures were found. He was treated with Liposomal AmB and fluconazole but passed away without resolution of the infection after 10 months of therapy. | **7** |
| UK | Case report | 32-year-old immunocompetent female research chemist contracted *C. immitis* from the subcultures she was working with resulting in pulmonary CM in which she had a full recovery without any antifungal intervention. | **17** |
| France | Case series | Epidemiology paper reporting the incidence of CM in France. Eight patients with TB and a positive screening for CM. | **18** |
| Italy | Case report | 38-year-old immunocompetent male diagnosed with pulmonary CM after returning from USA, CM confirmed via microscopy and culture. The patient had amelioration. (Treatment not described) | **19** |
| Netherlands | Case report | 39-year-old immunocompetent male contracted pulmonary CM during a road trip to Southern parts of USA. He was initially given penicillin, subsequently symptoms worsened resulting in surgical intervention. | **20** |
| Norway | Case report | 25-year-old immunocompetent male soldier who trained in Texas for seven months, during training he exhibited cold like symptoms and was subsequently given penicillin. Upon return, he was admitted to hospital due to a pulmonary cyst and was diagnosed with pulmonary CM. He had stable disease, which was asymptomatic, no treatment was administered. | **21** |
| Netherlands | Case report | 45-year-old male diagnosed with pulmonary CM after returning from Western USA. (Risk factors, treatment, and outcome not described) | **22** |
| France | Case report | 50-year-old immunocompetent doctor who worked in Venezuela and travelled in North America and Europe; on his visit to California, he developed flu- like symptoms after 2 weeks and developed a skin rash. One month later a lesion appeared on the hand which was treated with AmB. (Sex and outcome not described). | **23** |
| Netherlands | Case report | 23-year-old immunocompetent male with a case of pulmonary CM after a visit to Texas for military training. Initially treated with antibiotics with negligible effect and was then treated with AmB which also had little effect leading to surgical intervention and re-starting on AmB. The patient recovered from his infection. | **24** |
| Poland | Case report | A case of pulmonary CM in which the patient made a full recovery whilst on antibiotics. (Age, sex, unknown) | **25** |
| Italy | Case report | 43-year-old immunocompetent male who developed pneumonia with oral involvement and lymphadenopathy after visiting Venezuela. The patient was treated with AmB which led to improvement. | **26** |
| Germany | Case report | 25-year-old immunocompetent female who had returned from visiting the US, initially diagnosed with TB and was treated with antibiotics. Afterwards, skin tests were performed to diagnose the patient with CM. The patient required resection of her lung. | **27** |
| Russia/USSR | Case series | Identified 35 diagnosed patients within a single hospital specialised in rare mycoses in Moscow. Description of one case where CM was identified in the pelvic region. CM was characterised by infiltrated abscesses with multiple fistulas, for most cases symptoms lasted 15-20 years. | **28** |
| Finland | Case report | 2 patients with pulmonary CM.  Case 1: was a 21-year-old pregnant female who visited Arizona. After diagnosis she received a resection of the lung and AmB for ~ 2 months (1 month was for prophylactic use - post operatively).  Case 2: 24-year-old male nurse in England, initially diagnosed with TB and thus prescribed antibiotics to which he did not respond, subsequently had a skin test which was positive for *Coccidioides* spp. and was treated with AmB. Both patients were asymptomatic at last check-up. | **29** |
| Republic of Ireland | Case report | 24-year-old male with Hodgkin's disease for which he was treated with chemotherapy prior to a trip to Arizona. Upon return he received further chemotherapy due to a deterioration in his condition, patient later died, a post-mortem detected *Coccidioides immitis.* | **30** |
| Switzerland | Case report | A 41-year-old immunocompetent female from Arizona who came to Switzerland on holiday and presented with pulmonary CM. She was initially treated with antibiotics due to a suspicion of TB, but her disease progressed. Afterwards, a lobectomy was performed before confirming CM. | **31** |
| Sweden | Case report | A 45-year-old female diagnosed with CM who was unable to resolve their infection. (Risk factor, travel history, and treatment unknown) | **32** |
| Italy | Case report | A 68-year-old female who had no report/history of country where the infection was acquired, diagnosed via histology with pituitary granuloma. She made a full recovery and had surgery in conjunction with ketoconazole treatment. | **33** |
| Czech Republic | Case report | A case of pulmonary CM after returning from a visit to Arizona, USA. The patient was diagnosed via serology. (Age, sex, treatment, and outcome unknown). | **34** |
| Denmark | Case report | A patient with Hodgkin’s lymphoma was diagnosed with CM post-mortem. They presented with pulmonary CM which spread to the liver and CNS causing meningitis. (Age, sex, and treatment unknown). | **35** |
| Germany | Case report | 25-year-old male American soldier in Germany who presented with a chest wall abscess. (Treatment and outcome unknown). | **36** |
| Sweden | Case report | 43-year-old immunocompetent male with no travel history to an endemic area who developed a kidney infection. (Treatment and outcome unknown). | **37** |
| Hungary | Case report | 61-year-old immunocompetent male, originally from Arizona who presented with pulmonary CM whilst in Hungary. He was asymptomatic and was treated with ketoconazole reporting a full recovery. | **38** |
| Switzerland | Case series | One patient with concomitant CM and HIV. (Age, sex, travel history, treatment, and outcome not described). | **39** |
| Sweden | Case report | 68-year-old immunocompetent male who had previously visited Arizona. CM was confirmed via culture presented with pneumonia, the patient did not receive any interventions and was lost to follow up. | **40** |
| Switzerland | Case report | 47-year-old female who had previously visited Mexico and Los Angeles with sweet's syndrome presented with pulmonary and cutaneous CM. Skin lesions led to biopsy and diagnosis of CM. Patient was lost to follow up. | **41** |
| Germany | Case Report | Epidemiology of fungal infections in a single hospital during 1970-1993. An immunocompromised patient was identified to have cerebral mycosis due to CM. The patient was unable to resolve their infection and passed away. (Age, sex, and treatment not described) | **42** |
| Hungary | Case report | 21-year-old immunocompetent male student who studied in USA. They presented with a pulmonary infection and had surgical intervention leading to diagnosis of CM leading to the initiation of oral itraconazole for 6 months. Patient recovered from infection | **43** |
| Sweden | Case report | 65-year-old immunocompetent female smoker who spent 2 years in Arizona, USA. Upon return, she presented with a localised pulmonary infiltrate. Following this she had a thoracotomy for a suspected tumour, which was then identified as CM. No antifungals were given, and she reported a full recovery. | **44** |
| France | Case report | 46-year-old immunosuppressed male with HIV, originally from Colombia presented with a pulmonary infection. He had concomitant disease of histoplasmosis and CM based on blood culture and BAL. Patient was treated with cotrimoxazole and fluconazole for 10 days and then discharged. After 10 days the patient was readmitted with dry cough, weight loss and fatigue - inflammatory syndrome which was due to CM and histoplasmosis coinfection. After 2 months the patient was still apyrexic and gained 3 kg. (lost to follow-up). | **45** |
| France | Case report | 44-year-old immunocompetent male diagnosed with pulmonary infection after returning from Death Valley in California, USA. He was treated with itraconazole and recovered. | **46** |
| France | Case report | 63-year-old immunocompetent female who previously travelled to California, USA, presented with pulmonary CM. Initially suggested a diagnosis of community acquired pneumonia and then TB. Blood test and cultures from BAL, and molecular analysis led to diagnosis of CM. She was treated with posaconazole for 3 months leading to clinical resolution. | **47** |
| Germany | Case report | 52-year-old immunosuppressed female with pulmonary sarcoidosis who previously travelled to Arizona, USA. She developed pulmonary infiltrates and cutaneous granulomatous abscesses and same presentation when relapsed. Initially treated with AmB but discontinued (nephrotoxicity). Then took itraconazole for 5 months and had resolution at that time. She subsequently relapsed and was given Posaconazole (400 mg BID) and had a full recovery. | **48** |
| Poland | Case report | 38-year-old immunocompetent male who travelled to Arizona, USA and developed a pulmonary infection upon return. The patient was treated with NSAID for symptomatic treatment, no antifungal treatment was prescribed. The patient was unable to resolve his infection but had stable disease. | **49** |
| France | Case report | 65-year-old diabetic male who previously had disseminated infection with CNS involvement after surgery for the placement of a ventricular cardiac shunt. After returning from California, USA he initially presented with cutaneous lesions and then the infection disseminated causing meningitis. The patient was treated with fluconazole and survived but requires life-long treatment. | **50** |
| Switzerland | Case report | 61-year-old male with CLL who presented with a nodule in lower left lobe of the lung and developed pneumonia two weeks after his visit to Arizona, USA. He was diagnosed after a microscopy, culturing, and histology of tissue after a lung resection. He was able to resolve his infection spontaneously. | **51** |
| Belgium | Case report | 34-year-old immunocompetent male smoker presented with a persistent cough which was diagnosed as CM after returning from Arizona, USA, leading to the excision of a pulmonary lesion. It was initially thought he had a tumour until the excision which led to culturing of the pathogen. The patient recovered from the infection. | **52** |
| Netherlands | Case report | 35-year-old immunocompetent male who presented with pulmonary CM 3 weeks after returning from California, USA. He was initially treated with antibiotics prior to CM diagnosis and after he was treated with itraconazole. The patient survived, but unclear whether he was able to resolve his infection. | **53** |
| Netherlands | Case report | 58-year-old immunocompetent male with a previous history of mitral valve prolapse and atrial fibrillation. He presented with pulmonary (reticulonodular aberrations) enlarged lymph nodes after returning from California, USA. He was initially treated with a cephalosporin before diagnosis of CM. Fluconazole was then initiated, but did not respond to the treatment and then switched to ketoconazole. The patient had a full recovery. | **54** |
| France | Case report | 57-year-old immunocompetent female, previous smoker presented with febrile pneumonitis (pulmonary), arthralgia and erythema nodosum after returning from Arizona, USA. Amoxicillin and azithromycin were initiated for 8 days and symptoms disappeared; returned 6 weeks later: subsection of the lesion for diagnostic, and identification of cocci but no treatment as she was asymptomatic. | **55** |
| Italy | Case report | 48-year-old male with HIV who presented with lymphadenopathy after returning from USA. He was diagnosed via histology and treated with fluconazole leading to a full recovery. | **56** |
| Italy | Case report | 28-year-old immunocompetent male who travelled to Arizona, USA. Diagnosed via serology, microscopy, culture from BAL. He presented with pulmonary CM (Multiple nodules) and an enlarged spleen with small areas of reduced density (Disseminated CM). He was treated with itraconazole 200 mg BID for 6 months and reported a full recovery. | **57** |
| UK | Case report | 56-year-old immunocompetent male smoker who lived in Arizona, USA and now resides in the UK. He presented with a pulmonary infection and was treated with fluconazole. He made a full recovery, however after follow-up was found to have 2.5 cm pulmonary nodule which persists. | **58** |
| Netherlands | Case report | 77-year-old immunocompetent female who previously travelled to California, USA and had a knee replacement. She presented with a pulmonary infection which then spread to the knee. The patient was treated with oral fluconazole at 800 mg/day for 4 months and then reduced to 400 mg/day. She reported a full recovery. | **59** |
| Belgium | Case report | Patient with an autoimmune disease presented with pulmonary infection which led to dissemination (Age, sex, treatment, and outcome unknown) | **60** |
| Italy | Case report | 49-year-old immunocompetent male travelling from Venezuela who presented with a pulmonary infection.CM infection was confirmed via serology and was treated with IV fluconazole (800 mg/day) for 2 weeks and then 400 mg for 6 months and had a full recovery. | **61** |
| Turkey | Case report and literature review | 41-year-old immunocompetent male who previously travelled to Texas, Las Vegas, The Grand Canyon, and San Francisco. He presented with a pulmonary (white mucosal nodularity) infection and was treated with Itraconazole (100 mg) then switched to AmB (due to suspicion of blastomycosis) then back to itraconazole (200 mg). He made a full recovery. | **62** |
| Italy | Case report | 56-year-old immunocompetent female working in Argentina was admitted to hospital in Italy, diagnosed via microscopic detection, culture and histology after presenting with persistent erythematous papular plaque (skin infection). She was treated with itraconazole but was lost to follow-up. | **63** |
| France | Case report | 70-year-old female with spondylarthritis, anterior uveitis, obese, intercostal shingles, and hypertension who previously visited USA. She had a primary pulmonary infection which then spread to the CNS and developed meningitis. She was treated with fluconazole (800 mg/day) and afterwards liposomal AmB and made a full recovery. | **64** |
| Republic of Ireland | Case report | 64-year-old immunocompetent male with benign prostatic hypertrophy and, mild impairment of liver function who previously travelled to USA. He developed a pulmonary infection and was treated with AmB, fluconazole (IV). He made a full recovery. | **65** |
| Spain | Case report | 74-year-old male with a knee replacement presented with a pulmonary infection whilst residing in California, USA. Upon moving to Spain, the infection was re-activated spreading to the prosthetic joint in his knee. He was treated with the placement of a joint spacer containing AmB. The patient was also on oral itraconazole for 6 months and had clinical improvement. | **66** |
| Turkey | Case report | 30-year-old immunocompetent male who previously worked in Arizona, USA for 4 months. He developed pulmonary CM in USA and was reactivated on his return to Turkey. He was treated with Piperacillin-tazobactam IV, and fluconazole 400 mg IV once daily were started. After being discharged from hospital he was on fluconazole for 6 weeks and remained asymptomatic and tested negative for coccidioidal antibodies. | **67** |
| Denmark | Case report | 32-year-old immunocompetent female who had previously visited Argentina, Guatemala, Nigeria, Zambia, and South Africa. She had disseminated CM with infection in the peritoneum and genitalia region following IVF. She was treated with 8 months of fluconazole with no improvement. Outcome of the patient is unknown, but at the time of the report she had not resolved her infection. | **68** |
| France | Case report | 79-yr-old immunocompetent French male who worked in USA and experienced a pulmonary embolism on a flight back to France. He had a pulmonary infection and had resection of the nodule. Outcome of the patient was not reported. | **69** |
| Spain | Case series | Reports of clinical data and prevalence of different fungal infections including CM in hospitals from '97-'14 which found 94 cases due to migration. Overall, 43 had isolated pulmonary disease, 13 meningitis, 7 cutaneous forms, 3 disseminated disease, 28 patients had no data. of which 7 patients died, 59 cases were discharged, and 3 patients were re-admitted. | **70** |
| Sweden | Case series | A retrospective study looking at the annual burden of fungal infections in Sweden via national registry data. One patient was identified to have been diagnosed with CM in 2016. (Age, sex, treatment, and outcome not described) | **71** |
| Lithuania | Case report | 31-year-old immunocompetent male who visited California, USA and developed a pulmonary infection upon return. Four months after, sputum samples were sent to Germany for analysis and came back with CM diagnosis. The patient was then treated with fluconazole and made a full recovery. | **72** |
| Spain | Case report | 39-year-old male who previously travelled to Arizona, Nevada, and California - the site of infection was on his left cervical lymphadenopathy. He was treated with oral fluconazole (400 mg/day) for 5 months, recovered and took reduced dose of fluconazole (200 mg/day) for prophylaxis. | **73** |

AmB: Amphotericin B; BAL: Bronchoalveolar lavage; BID: Bis in Die; CM: Coccidioidomycosis, CLL: Chronic Lymphatic Leukaemia; IV: Intravenous; IVF: *In Vitro* Fertilisation; NSAID: Nonsteroidal Anti-Inflammatory Drug; USA: United States of America
